# Calcaneal tuberosity avulsion fracture fixation: a technical note and results on cases of patients

**DOI:** 10.3389/fsurg.2026.1825111

**Published:** 2026-10-16

**Authors:** PengKun Yu, Fei Zhao, Qiang Xu, Yu Wu, Min Gong

**Affiliations:** 1 Department of Orthopedics Chengdu, Wenjiang Traditional Chinese Medicine Hospital of Chengdu, SIchuan, China; 2 Department of Orthopedic Surgery, Affiliated Sport Hospital of Chengdu Sport University, Chengdu, Sichuan, China

**Keywords:** Achilles tendon, bunnell, calcaneal tuberosity avulsion fracture, minimally invasive technique, sural nerve.

## Abstract

**Objective:**

Calcaneal tuberosity avulsion fractures are more common in elderly osteoporotic populations. According to the Beavis classification, they are divided into 3 types. Types I and III have small bone fragments that are difficult to fix with screws, and traditional open surgery has a high rate of skin necrosis and poor wound healing. This article proposes a modified Bunnell suture combined with calcaneal tunnel fixation technique that can flexibly switch between minimally invasive and open approaches, and explores its short-term clinical efficacy.

**Methods:**

From January 2024 to February 2026, 2 patients with calcaneal tuberosity avulsion fractures were enrolled. One patient with Beavis type II + I underwent open surgery, and one patient with Beavis type II underwent a minimally invasive procedure. Postoperatively, the Burwell-Charnley criteria were used to assess the quality of fracture reduction. At 6 months postoperatively, the AOFAS ankle-hindfoot scale was used, and the maximum plantarflexion angle and dorsiflexion angle of the ankle joint were recorded. Follow-up recorded incision, fracture healing, and complications, with an average follow-up of 1 year.

**Results:**

Both patients achieved anatomical reduction postoperatively, and all achieved bony union at 3 months postoperatively without loss of reduction. At 6 months postoperatively, the AOFAS scores were 83 and 96, respectively, and both the maximum plantarflexion and dorsiflexion angles were both within the normal range. Only the patient who underwent open surgery and had diabetes and obesity experienced delayed wound healing, which healed after regular dressing changes for 1 month. The minimally invasive case did not develop complications related to soft tissue, sural nerve, or Achilles tendon.

**Conclusion:**

The modified Bunnell suture combined with calcaneal tunnel fixation technique may help balance rigid fixation and soft tissue protection. The minimally invasive approach appears to lower the risk of incision-related complications in the present series. This technique may offer a flexible alternative for selected cases of Beavis calcaneal tuberosity avulsion fractures, especially suitable for minimally invasive fixation of types I and III fractures, and may also act as an auxiliary fixation method for type II fractures. However, the sample size of this study is small, and long-term efficacy still needs to be verified by large-sample controlled studies.

## 1 Introduction

Calcaneal tuberosity avulsion fractures are common in elderly patients with osteoporosis, obesity, and those with diabetes, mostly caused by sudden traction of the Achilles tendon resulting in avulsion injury of the calcaneal tuberosity, accounting for about 3% of all calcaneal fractures (1). When this type of fracture is significantly displaced, it can easily lead to abnormally increased local soft tissue tension, secondary flap ischemia and necrosis, and in severe cases, exposure of the Achilles tendon and bone at its insertion may occur.

The classic Beavis classification divides these injuries into three types of extra-articular avulsion fractures: Type I is a sleeve-like cortical bone avulsion fracture, Type II is a beak tuberosity fracture, and Type III is a sub-synovial avulsion fracture of the posterior tuberosity (2). Under the standard classification, the fracture lines of all three types do not invade the subtalar joint; therefore, typical calcaneal tuberosity avulsion fractures themselves do not involve intra-articular injury. Only Beavis type II has a special variant subtype, in which the oblique fracture line can break through the posterior subtalar articular cartilage surface anteriorly, making it the only tuberosity avulsion injury that can be combined with intra-articular involvement. Once the type II variant fracture destroys the subtalar articular cartilage, traumatic subtalar osteoarthritis will develop in the long term—this disease is a secondary osteoarthritis induced by articular cartilage injury, with typical pathological features of cartilage defects, subchondral bone sclerosis, osteophyte formation, and joint space narrowing. Patients have persistent heel pain during weight-bearing walking, conservative treatment has limited effect, and severe cases require subtalar arthrodesis. Therefore, surgical treatment is recommended for displaced type II variant fractures with joint involvement.

Lag screw is the mainstream fixation method for type II fractures, but for types I and III fractures, the avulsed bone fragments are small in size, making it difficult for screws to achieve reliable fixation and unable to effectively counteract the continuous traction stress of the Achilles tendon. Currently, clinical options also include tension band fixation, suture anchors, suture bridge, side-locking loop suture (SLLS), and Tightrope technique, but each of these procedures has its limitations: tension band easily irritates local soft tissue and induces skin necrosis; anchors have a risk of loosening in osteoporotic bone; some suture techniques may cause secondary chronic plantar pain postoperatively (3–5); and at the same time, it is difficult for various procedures to properly fix the small avulsed fragments of types I and III. Providing sufficient fixation strength, allowing early weight-bearing, avoiding skin necrosis, and protecting the blood supply of the Achilles tendon are the core principles of treatment for this fracture (6).

In view of the shortcomings of existing procedures, this study proposes an innovative repair technique that can flexibly choose a minimally invasive or open surgical approach according to the actual fracture situation, namely the modified Bunnell suture combined with calcaneal tunnel fixation technique, and confirms the short-term efficacy of this technique through 2 clinical cases, aiming to provide a more optimized surgical treatment plan for calcaneal tuberosity avulsion fractures.

## 2 Materials and methods

The same orthopedic surgeon at our institution treated 2 patients with calcaneal tuberosity avulsion fractures using this innovative technique from January 2024 to February 2026. Preoperatively, we collected statistics on the patients' body mass index, fracture classification, and diseases, and performed surgical treatment on the second day. The final follow-up was conducted at 12 months (48 weeks) postoperatively. Postoperative records of related complications, and review of imaging data (at 4, 8, and 12 weeks postoperatively) were collected. At the final follow-up, the range of motion of the ankle and foot function was recorded. The AOFAS was used to evaluate the final outcome. The Burwell-Charnley radiological reduction criteria were used to evaluate the quality of postoperative fracture reduction (7).

At the final follow-up, the maximum dorsiflexion angle and maximum plantarflexion angle of the affected ankle joint were precisely measured in all patients. Using the physiological range of motion of the normal adult ankle joint as the reference baseline (dorsiflexion 0°–20°, plantarflexion 0°–45°), the recovery level of joint function of the affected limb was quantitatively evaluated.

At the final follow-up, the AOFAS ankle-hindfoot scale was used (8), which consists of three dimensions: pain, function, and appearance. The specific sub-scores include the following items: 1. Pain (40 points): including assessment of pain aggravation and relief, timing and severity of pain, etc. 2. Function (50 points): including assessment of gait, range of motion, muscle strength, and activity limitations, etc. 3. Appearance (10 points): including assessment of swelling, scar, deformity, and skin condition, etc. (higher score indicates better function). Excellent: 90–100, Good: 75–89, Fair: 50–74, Poor: below 50.

The Burwell-Charnley radiological reduction criteria were used to evaluate the quality of postoperative fracture reduction. Although this standard was initially mostly used for evaluation of reduction of Pilon fractures and complex ankle fractures, because of its clear quantitative indicators and suitability for evaluation of fracture reduction around limb joints, it can accurately determine the displacement and angulation of the fracture fragments of calcaneal tuberosity fractures, and is suitable for the needs of evaluating the reduction efficacy of calcaneal tuberosity avulsion fractures in this study. The fracture reduction radiological evaluation criteria (Burwell-Charnley) were used for postoperative assessment: anatomical reduction: no medial or lateral displacement or angulation of the fracture fragments, fragment displacement <2 mm; acceptable reduction: no obvious medial or lateral displacement or angulation of the fracture fragments, 2 mm < fragment displacement <5 mm (7).

## 3 Technical note

### 3.1 Preoperative detailed preparation

All patients underwent surgery on the second day after admission, in the prone position, and combined spinal-epidural anesthesia was selected to reduce the impact on respiration and circulation. This technique supports both minimally invasive and open approaches. Therefore, an open-surgery case was included and corresponding intraoperative images were shown to reflect the design features of flexible conversion between surgical approaches.

### 3.2 Establishment of bone tunnel

After determining the fracture location, a 2.5 mm diameter Kirschner wire was used to establish a bone tunnel at the midpoint of the line connecting the lower edge of the heel and the medial malleolus, and a high-strength suture was introduced with a puncture needle (Figure 1A); When establishing the bone tunnel in the calcaneus, injury to the tarsal tunnel should be avoided. The bone tunnel established with the Kirschner wire should be adequately reamed to ensure the integrity of the bone tunnel and avoid suture wear.

**Figure 1:**
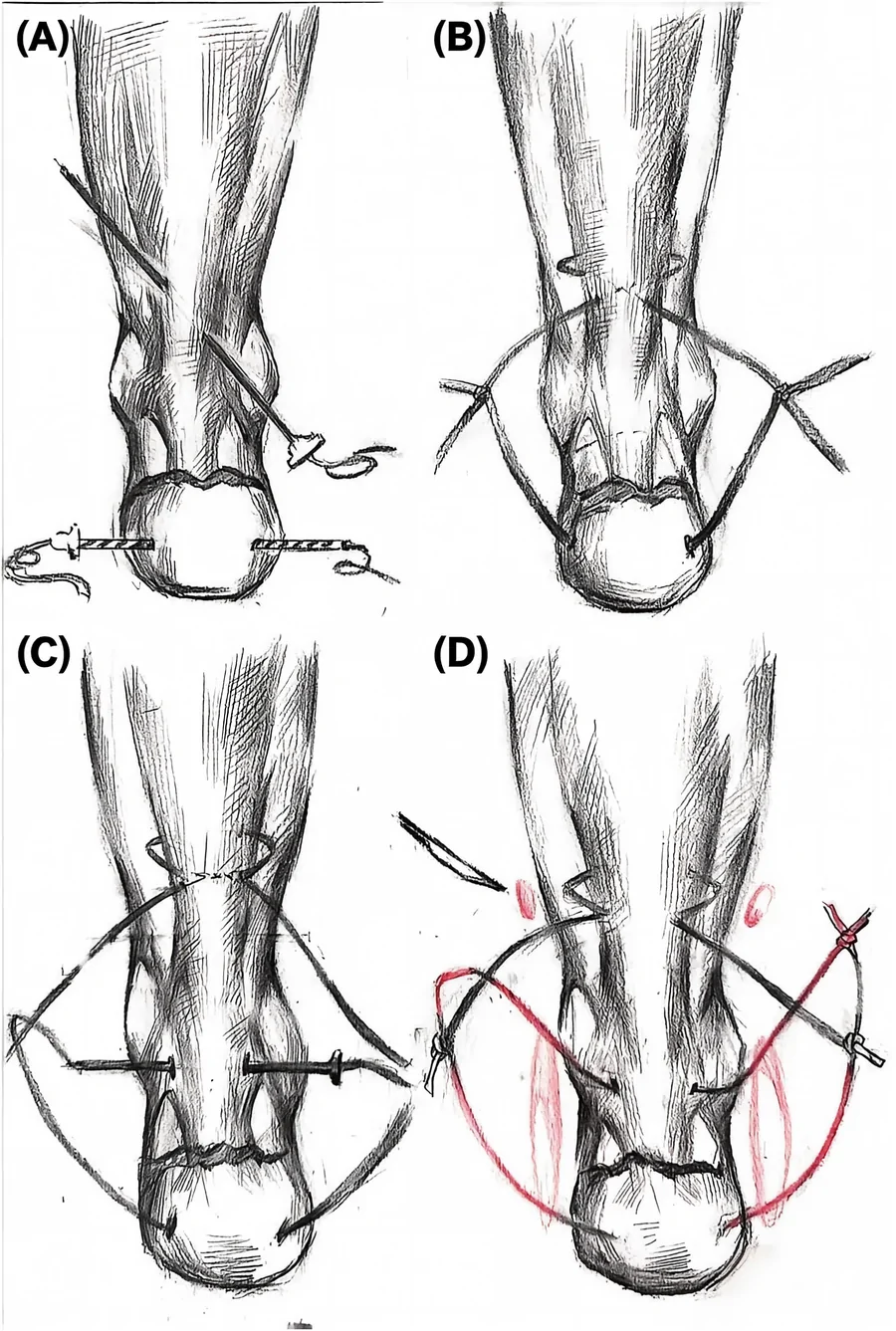
Surgical schematic diagram. **(A)** Use a 2.5 mm Kirschner wire to establish a bone tunnel and pass the suture using a puncture needle, and perform Bunnell suturing of the Achilles tendon (upper left); **(B)** perform Bunnell suturing to counteract Achilles tendon tension (upper right); **(C)** use an epidural puncture needle to penetrate the calcaneal junction (lower left); **(D)** tie knots to fix and reduce the fracture; the red oval represents the layout of the minimally invasive incisions (lower right).

### 3.3 Percutaneous minimally invasive suturing and repair of the achilles tendon

Use the percutaneous minimally invasive Bunnell technique to suture the Achilles tendon, ensuring stable and secure traction of the Achilles tendon; the suture from the calcaneal bone tunnel and the percutaneous Achilles tendon suture are tied bilaterally to adjust tension, ensuring the calf triceps compression test is negative (Figure 1B). When performing minimally invasive suturing of the Achilles tendon, the course of the sural nerve must be marked preoperatively (the line connecting the midpoint of the line between the tip of the lateral malleolus and the Achilles tendon to the midpoint of the popliteal fossa). On the premise of avoiding injury to this course, perform Bunnell suturing at the proximal end of the Achilles tendon (4). After suturing, ensure the suture is stable and does not slip, and check the Thompson test (Figure 2).

**Figure 2:**
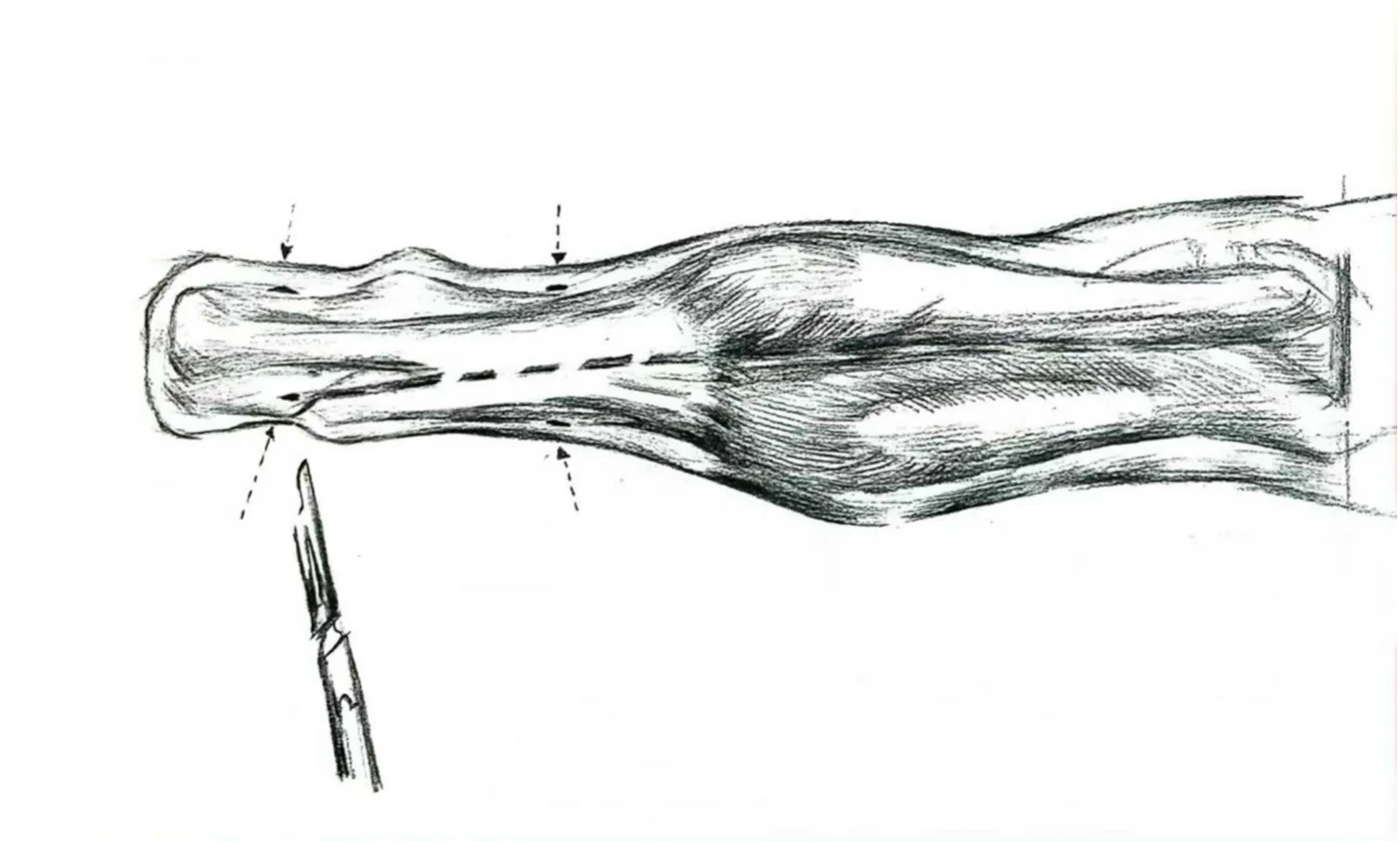
Course of the sural nerve.

### 3.4 Fracture reduction technique

When performing initial reduction of the avulsion fracture at the insertion point, a suture can be introduced at the Achilles tendon insertion, i.e., the tendon-bone junction, to pull the bone fragment downward to assist reduction, and Kirschner wires are used percutaneously to fix the bone fragment (alternatively, a posterior midline small incision can be used for direct visualization to reduce the fracture and fix it with Kirschner wires). The fracture reduction is checked under fluoroscopy (Figure 3).

**Figure 3:**
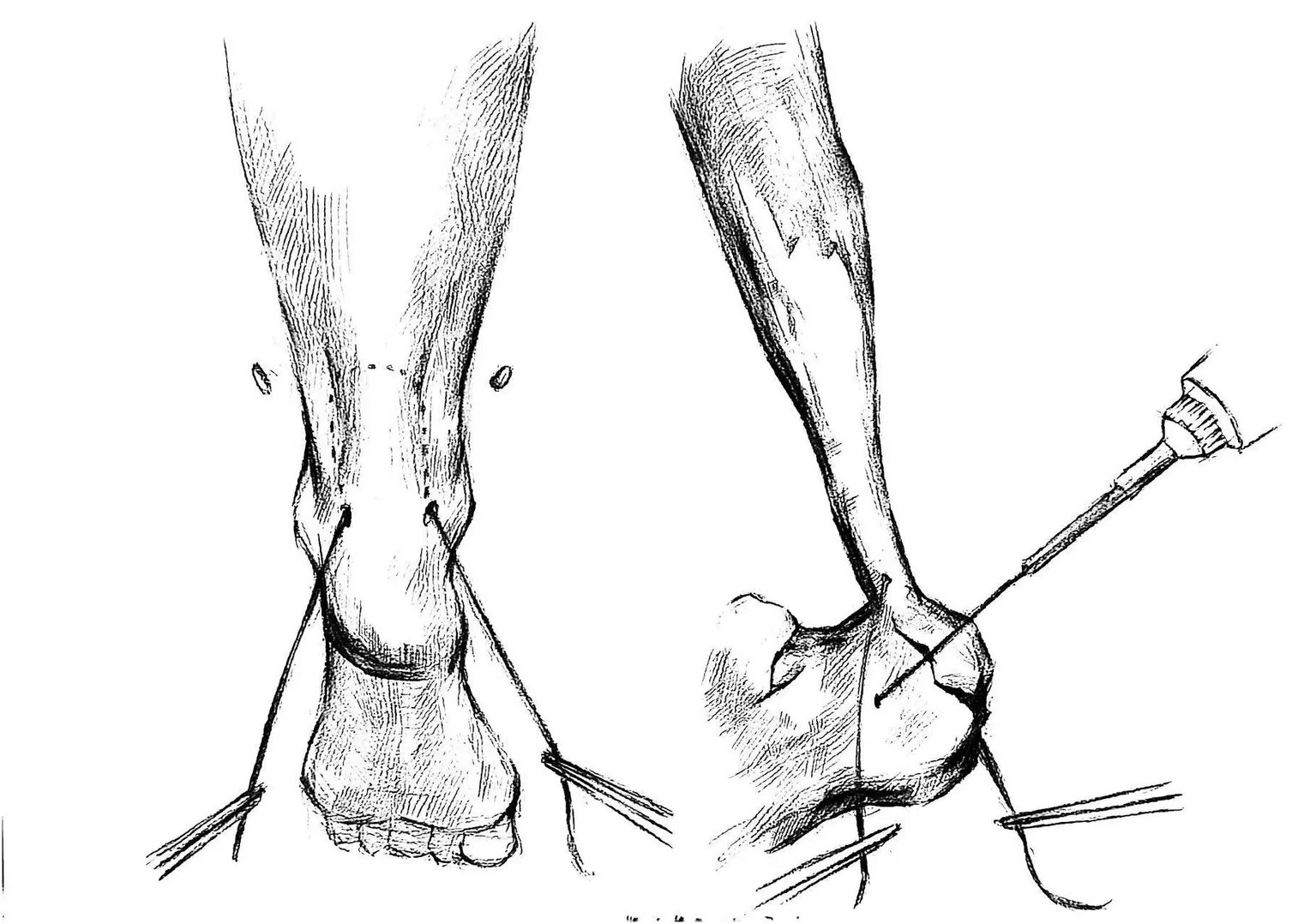
Assisted reduction technique. **(A)** Reduction assisted by the minimally invasive technique (left); **(B)** Kirschner wire fixation following reduction (right).

### 3.5 Knot tying for fracture fixation

Performing passing suture at the tendon-bone junction can provide stable fixation for smaller bone fragments (Figure 1C). All knots are tied beside the incision to reduce the risk of suture reaction, and absorbable high-strength sutures were selected for this surgery (Figure 1D).

### 3.6 Technical challenges and management

Iatrogenic sural nerve injury is the main complication risk associated with percutaneous Achilles tendon suturing. Preoperative marking of the sural nerve pathway to avoid injury to this nerve is an effective preventive measure. Secondly, poor reduction of the bone fragment is another technical limitation of this minimally invasive approach; this issue can be addressed by making a small midline posterior incision, combined with assisted reduction and temporary Kirschner wire fixation. When necessary, this technique can be converted to open surgery through a posterolateral S-shaped incision, but the main risk of the open approach lies in poor wound healing.

For type I and type III fractures, the minimally invasive approach is preferred. If the reduction of the bone fragment is unsatisfactory, assisted reduction techniques should be used first; conversion to open surgery should only be performed when reduction cannot be achieved. For calcaneal beak fractures, this technique combined with a limited incision can serve as an auxiliary fixation method to minimize the incidence of skin necrosis.

Postoperatively, a plaster splint was applied to fix the ankle joint in 30° of plantarflexion. The plaster splint was removed at 8 weeks postoperatively, and active flexion and extension exercises of the ankle joint were started. Short-term plaster immobilization can avoid complications such as disuse bone loss and ankle stiffness caused by prolonged external fixation in elderly osteoporotic patients. The affected limb was prohibited from weight-bearing until 3 months postoperatively, and after review at 3 months showing bony union of the fracture, the patient could begin progressive walking training, with normal weight-bearing gait restored at 6 months postoperatively.

## 4 Case illustration

Patient 1 was a 63-year-old female with type 2 diabetes, caused by a fall while walking, with a body mass index of 32.6 kg/m^2^, diagnosed with Beavis type II + I fracture, and underwent open surgery with cannulated screw fixation supplemented by the above-mentioned innovative fixation technique to repair the avulsion fracture at the Achilles tendon insertion (Figures 4–8). At 2 weeks after discharge, the patient did not have the wound dressing changed or sutures removed, and resumed smoking after discharge, leading to skin ulceration; however, after dressing changes, it healed completely at 1 month postoperatively, and review within three months showed fracture healing, and the Achilles tendon fixation and repair were secure (Figures 9, 10).

**Figure 4:**
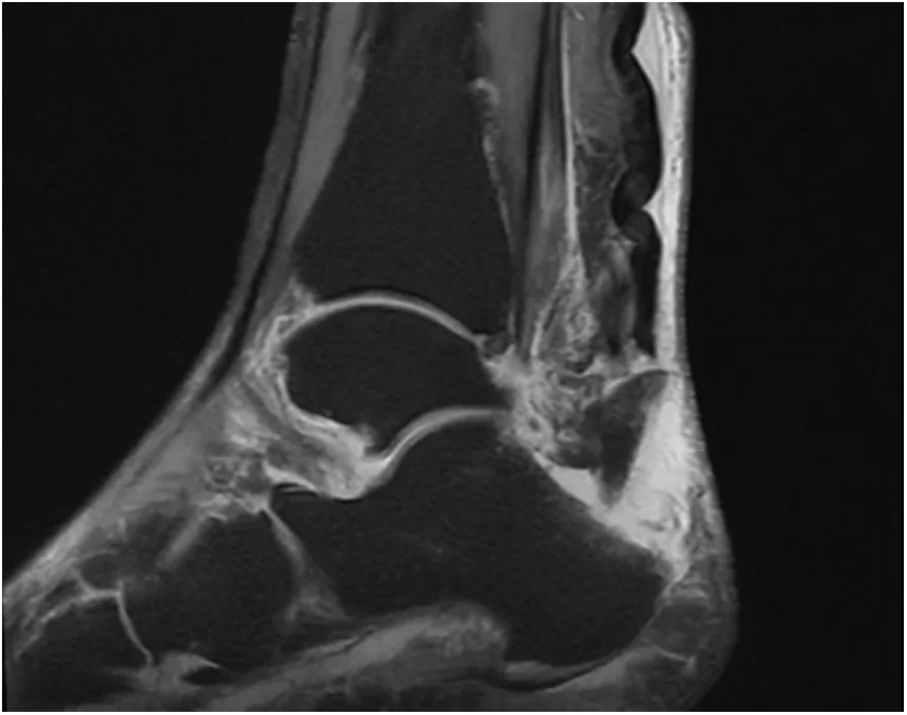
Preoperative MRI shows a beak fracture with avulsion fracture at the achilles tendon insertion.

**Figure 5:**
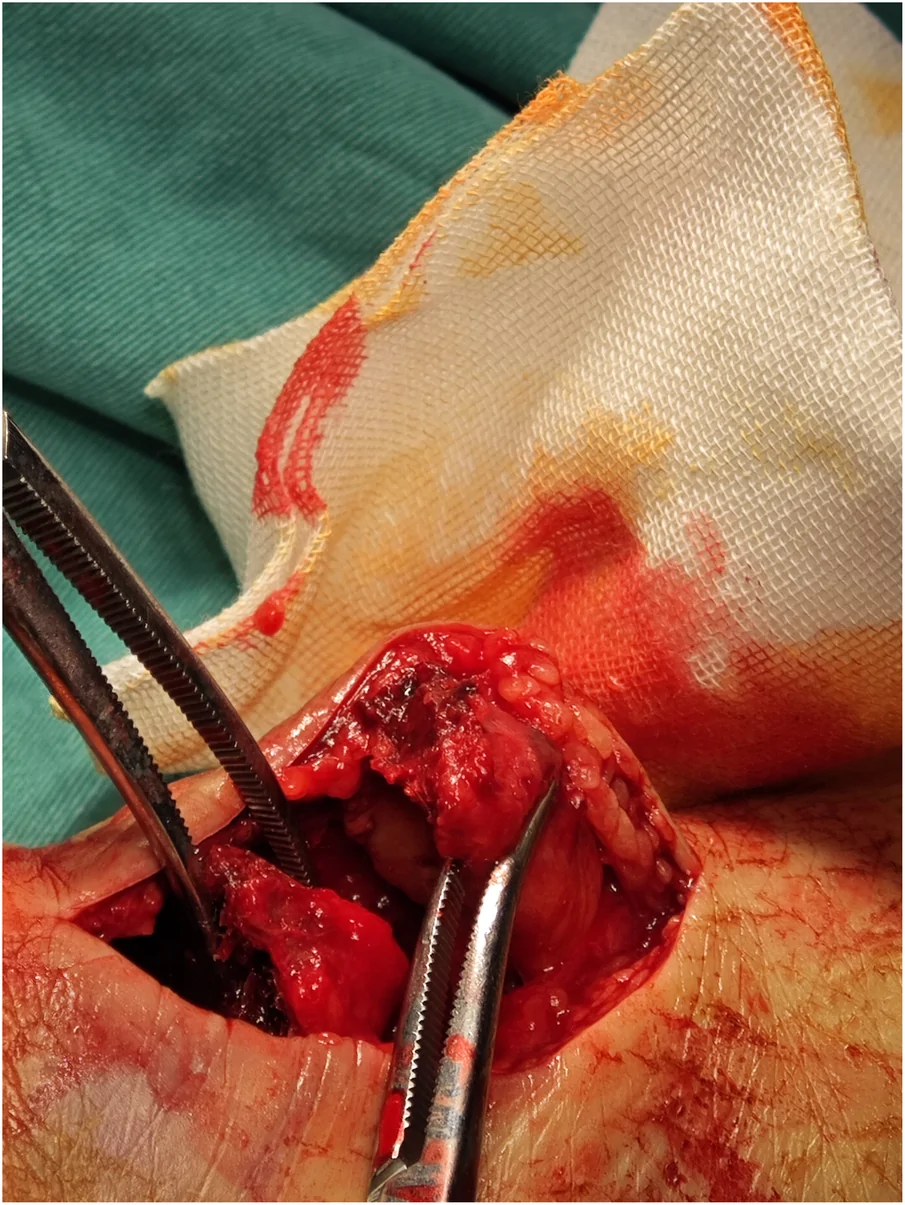
Intraoperative exploration confirmed Beavis type II + I fracture.

**Figure 6:**
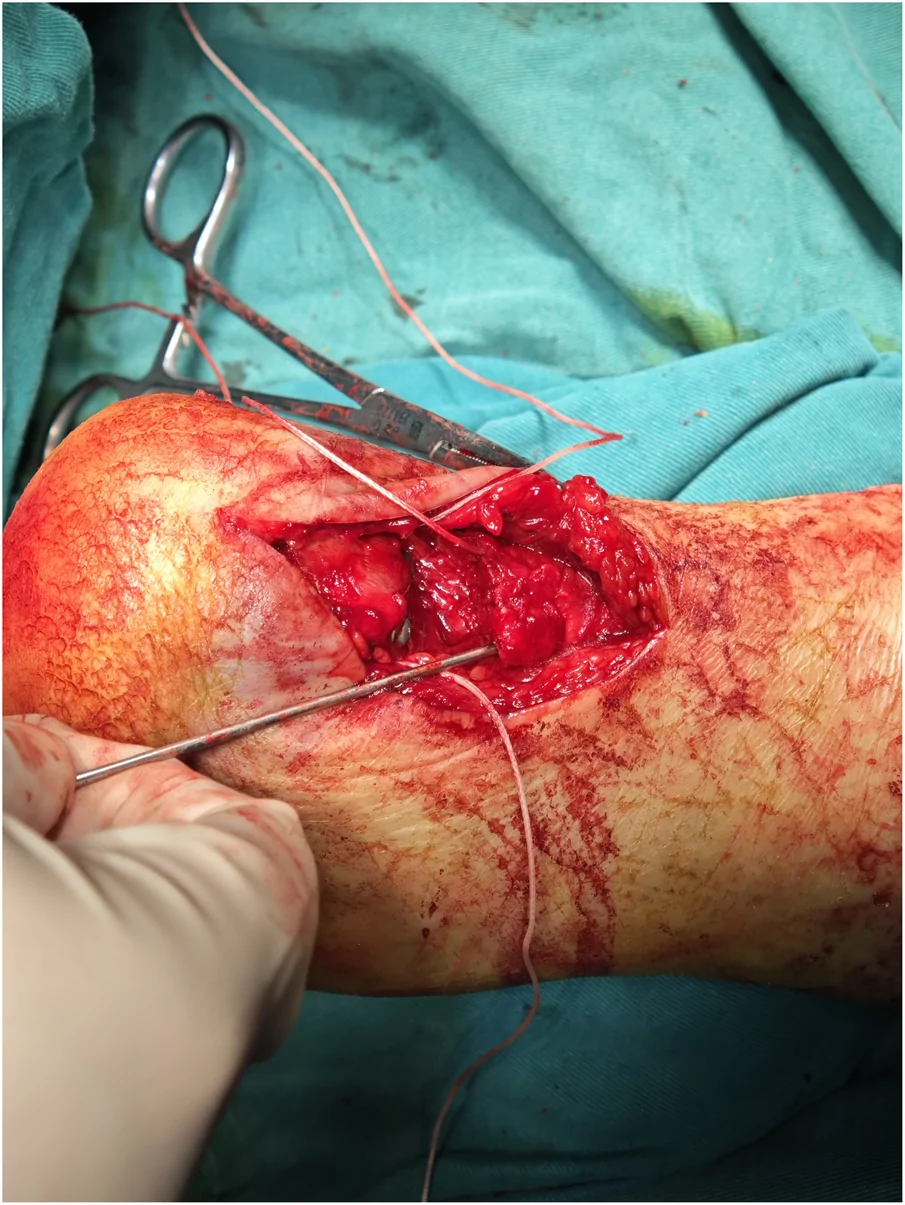
Intraoperatively, after fixing the “beak” fracture with cannulated screws, the above-mentioned innovative technique was used to establish a bone tunnel and repair the avulsion fracture at the Achilles tendon insertion.

**Figure 7:**
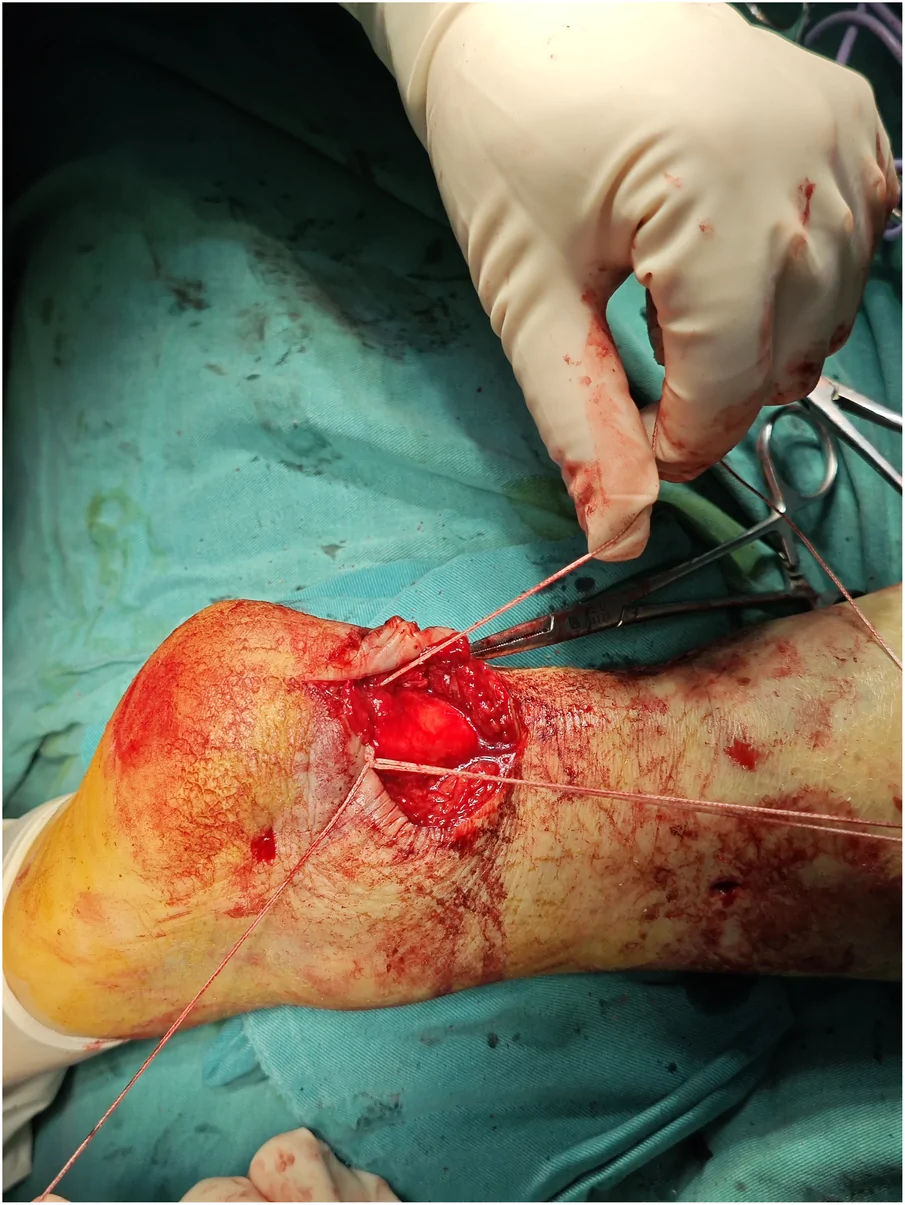
An epidural puncture needle was inserted into the tendon-bone junction at the Achilles tendon insertion, and after passing the suture, a secure knot was tied on the opposite side.

**Figure 8:**
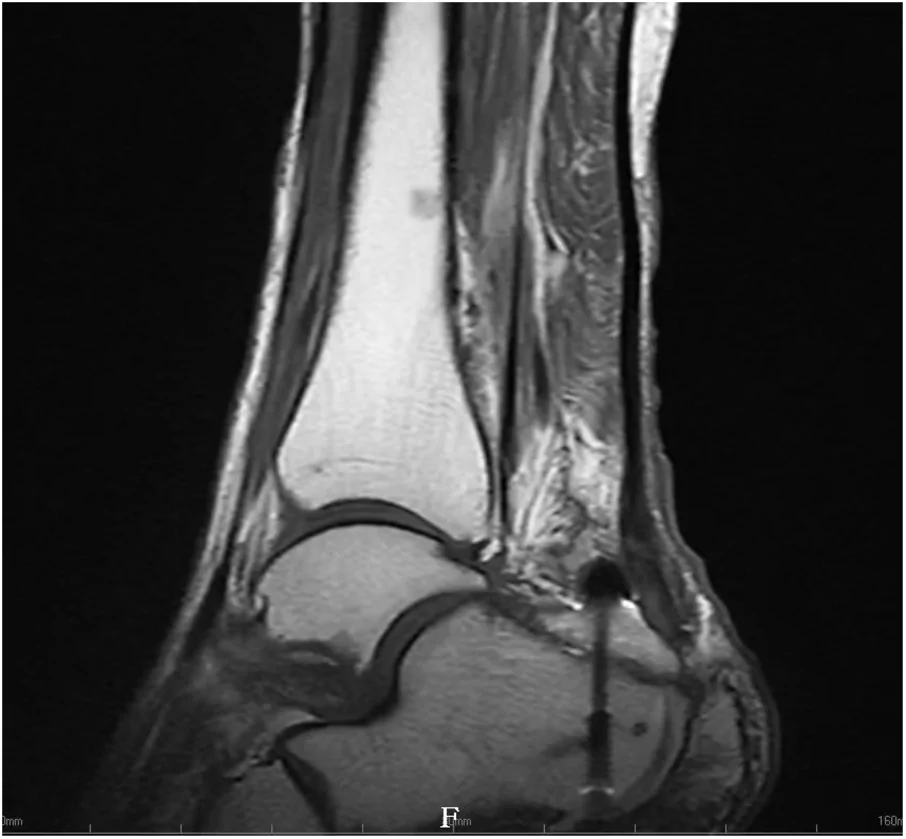
Postoperative MRI review shows continuity of the Achilles tendon and reduction of the beak fracture.

**Figure 9:**
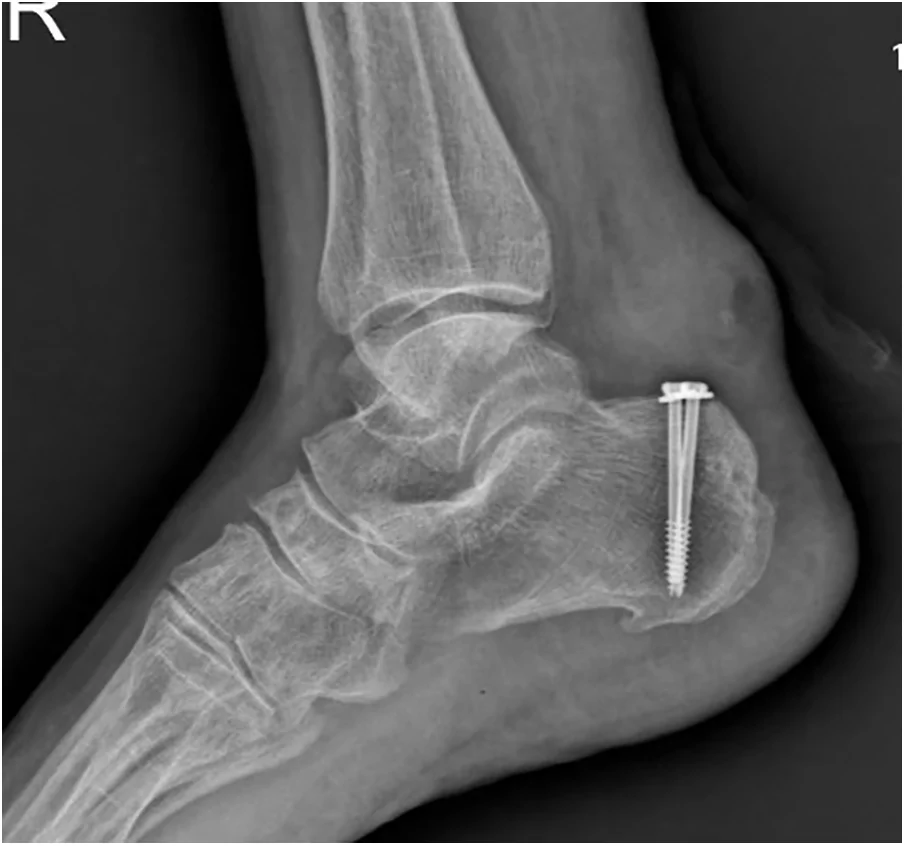
Radiographic follow-up at 6 months postoperatively.

**Figure 10:**
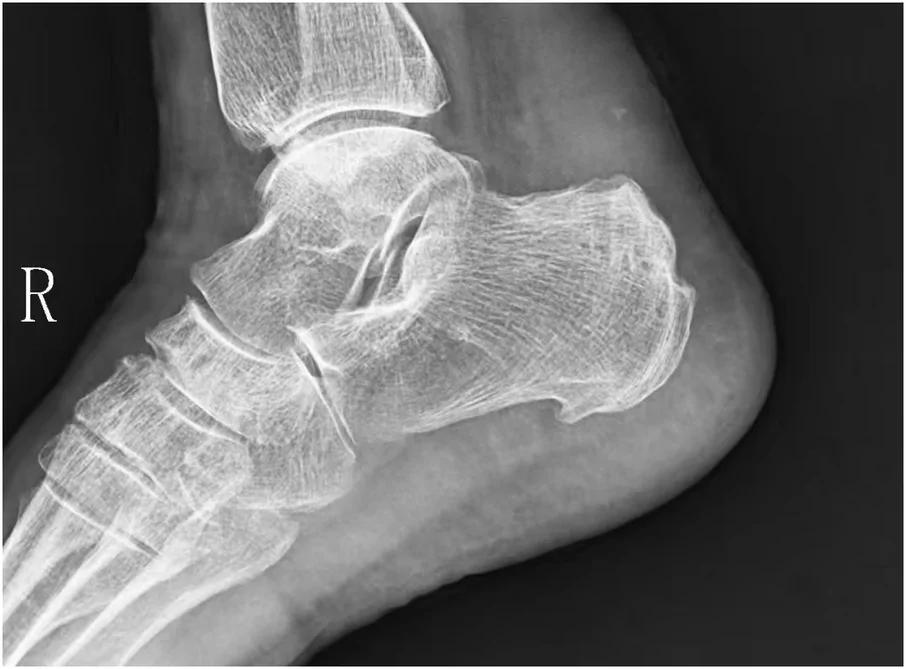
Removal of internal fixation device within 1 year postoperatively.

Patient 2 was a 52-year-old male with no other underlying diseases, caused by a fall while walking; body mass index was 28.4 kg/m^2^, no significant family history or surgical history; Patient 2 was diagnosed with Beavis type II fracture and underwent minimally invasive fixation (Figures 11–13); the wound healed well and the patient was discharged.

**Figure 11:**
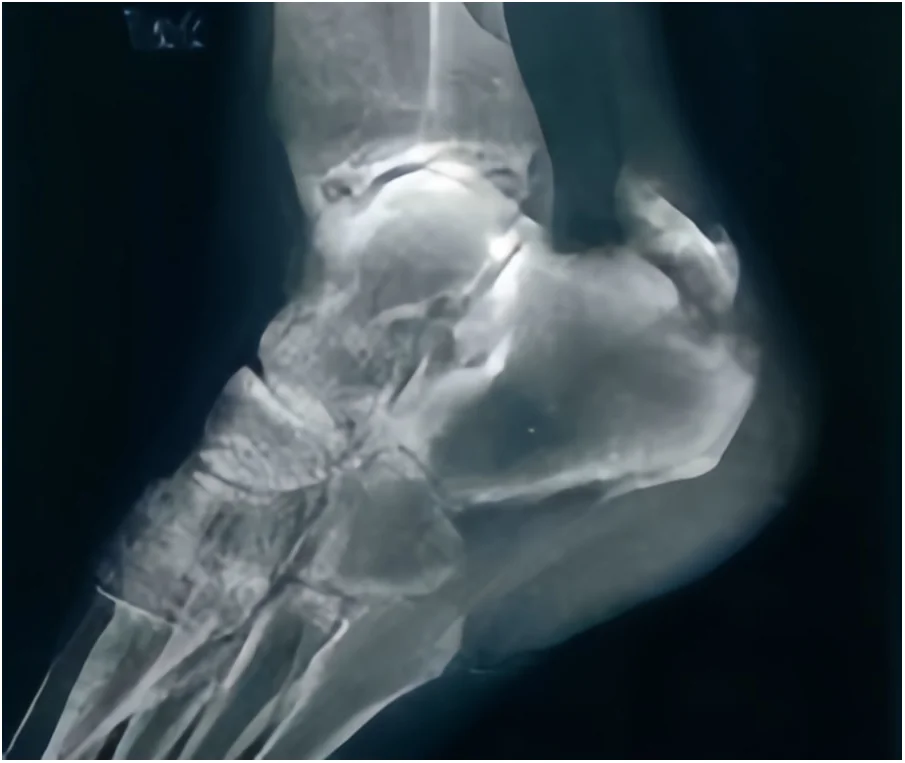
Preoperative Digital radiography (DR) of another patient shows calcaneal tuberosity avulsion fracture (Beavis I).

**Figure 12:**
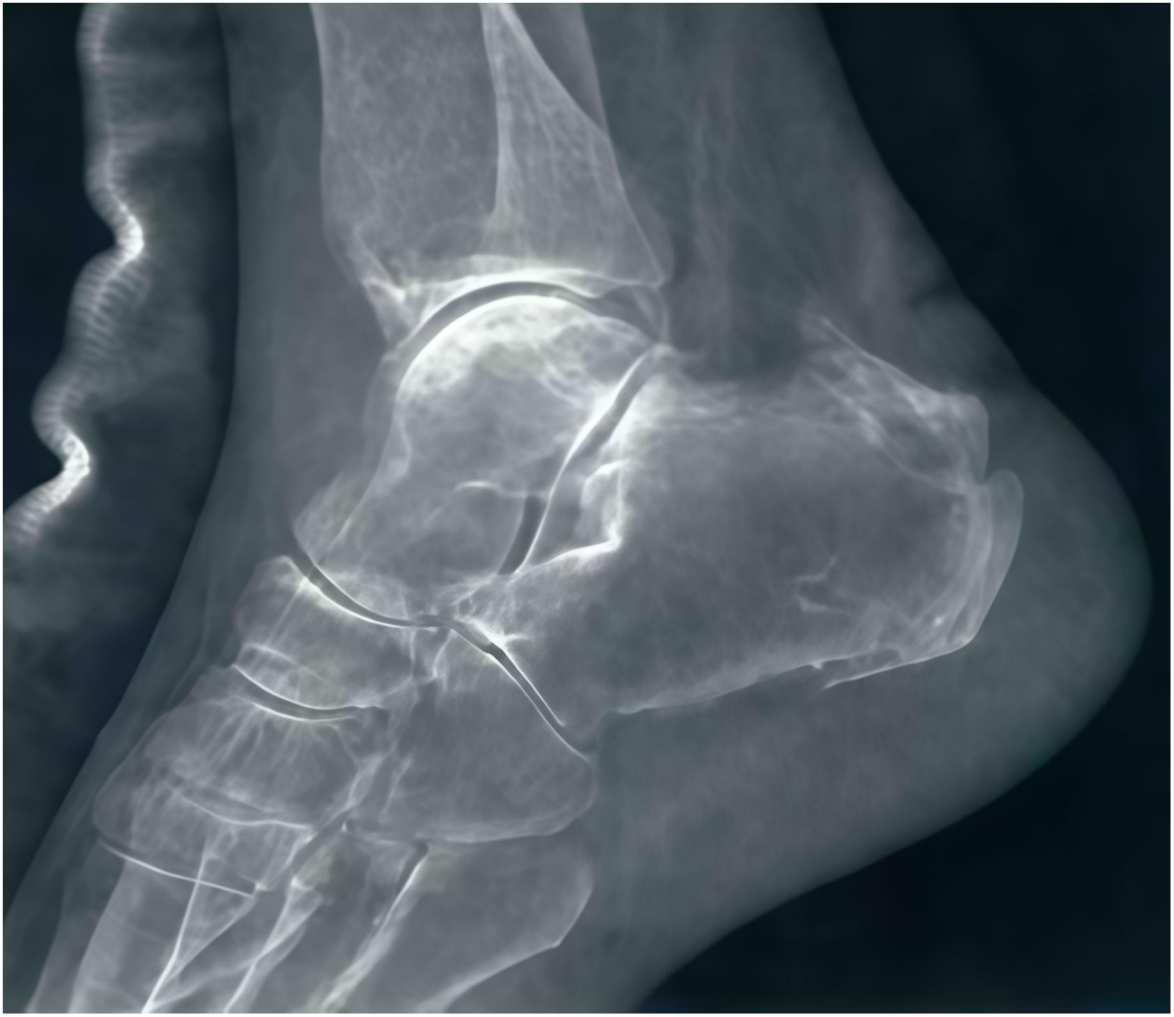
Successful reduction of the fracture using the minimally invasive technique and assisted reduction.

**Figure 13:**
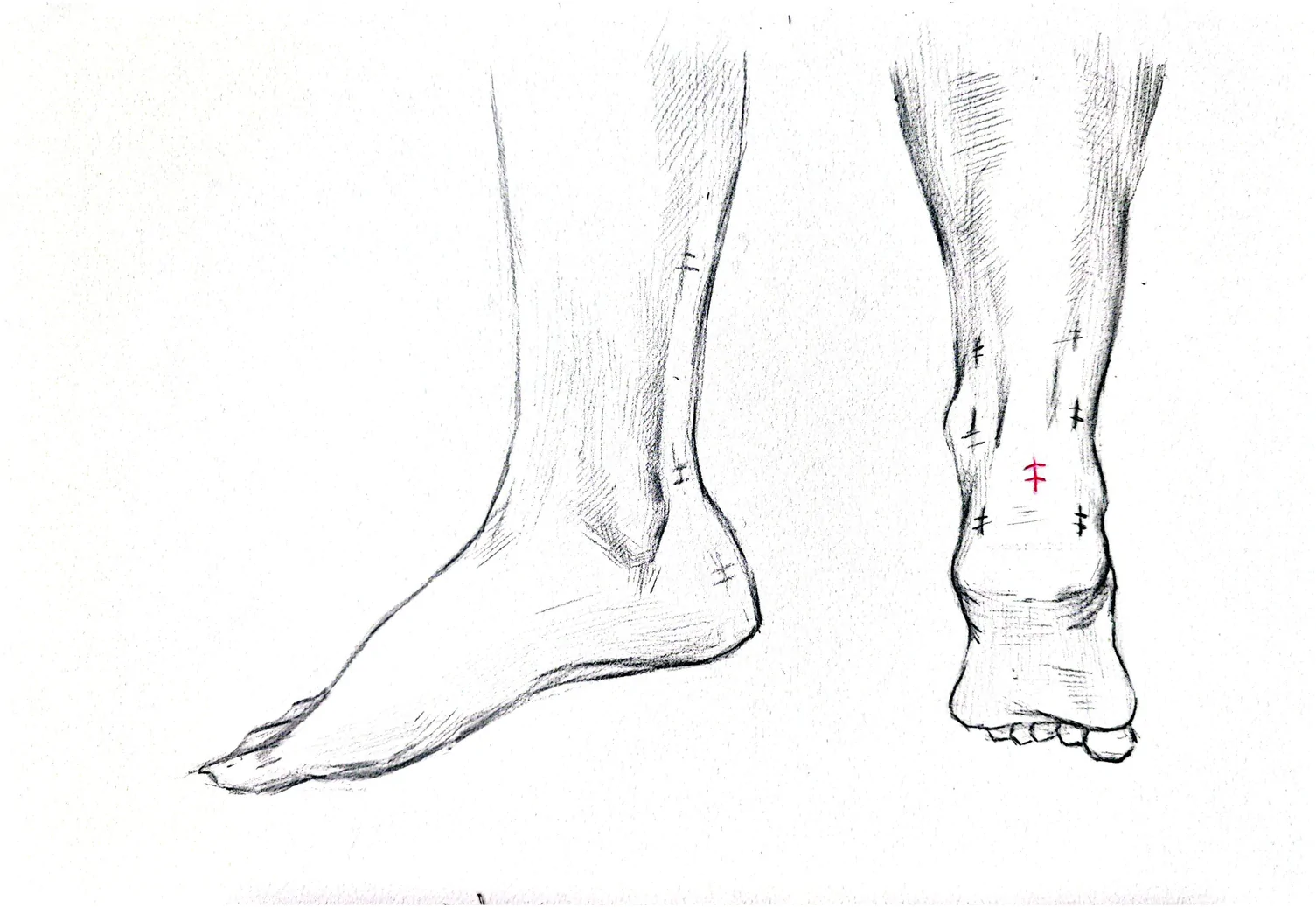
Incision description: layout of minimally invasive incisions; the red incision represents the posterior accessory incision.

Preoperative and postoperative MRI examinations were performed in the open surgery case, taking into account both fracture reduction and assessment of Achilles tendon soft tissue injury; the minimally invasive case was followed up entirely with DR plain films. The average follow-up period was 1 year. All patients achieved bony union without displacement of the fracture fragments at 3 months, and the ankle joint range of motion was unrestricted. All patients were able to return to their pre-injury daily activities (Table 1).

**Table 1:** The general information of patients.

Case	Gender	Age	Fracture type	Comorbidities	BMI	Complications	Postoperative AOFAS score (6 months)	Maximum plantarflexion/dorsiflexion angle	Burwell-Charnley evaluation
Patient 1	Female	63	Beavis II + I type	Type 2 diabetes	32.6 kg/m^2^	Poor wound healing	83	30.3°/14.1°	Anatomical reduction
Patient 2	Male	52	Beavis II	None	28.4 kg/m^2^	None	96	34.7°/16.2°	Anatomical reduction

## 5 Discussion

This study used this technique to treat 2 patients. Follow-up data indicated that all patients achieved anatomical reduction (Burwell-Charnley criteria), and all achieved bony union at 3 months postoperatively, with no fracture redisplacement. At 6 months postoperatively, the AOFAS ankle-hindfoot scores were 83 and 96, respectively. For the open surgery, the maximum plantarflexion angle of the affected ankle was 30.3° and dorsiflexion angle was 14.1°, while for the minimally invasive surgery, the maximum plantarflexion angle was 34.7° and dorsiflexion angle was 16.2°. Normal adult ankle reference values: dorsiflexion approximately 0°–20°, plantarflexion approximately 0°–45°. One patient with type 2 diabetes mellitus had poor wound healing postoperatively, which healed after 1 month of dressing changes, and no severe complications such as skin necrosis, Achilles tendon rupture, or nerve injury occurred. According to the above results, this technique provides reliable fixation; however, the issue of wound healing in open surgery still requires further study.

Literature reports that the reduction rate of traditional screw fixation is about 75%–85%, with a tendency for loosening and loss of reduction in osteoporotic patients, and the delayed union rate can reach 10%–15%. All cases in this group achieved anatomical reduction and bony union within 3 months, suggesting that modified Bunnell suture combined with calcaneal tunnel fixation has greater tension resistance and more reliable fixation, effectively reducing the risk of reduction loss and poor healing. For types I and III fractures, due to the small bone fragments and involvement of the Achilles tendon, the method of passing and fixing the suture at the tendon-bone junction can effectively fix the bone fragments that are difficult to fix.

In terms of function, literature shows that postoperative AOFAS scores for tension band fixation are mostly 70–85, and for anchor fixation 78–90, with some patients experiencing Achilles tendon contracture and limited heel raising (9). In this study, the AOFAS scores of the 2 patients were 96 and 83, respectively. Although the sample size of this article is small, the minimally invasive Bunnell suture has potential advantages in protecting the blood supply of the Achilles tendon and reducing tendon injury, but further verification with a large sample is still needed. In terms of complications, the incidence of skin complications with tension band fixation is as high as 64%, and the loosening rate of anchors in osteoporosis is 8%–12% (3). In this study, one case of open surgery had poor wound healing; literature indicates that the incidence of poor skin healing of open incisions in patients with diabetes and obesity (BMI > 30) can reach 20%–30%. The poor wound healing in this case was closely related to her advanced age, type 2 diabetes, BMI, and irregular postoperative smoking and dressing changes. The study of skin necrosis caused by open surgery itself still needs further discussion; the minimally invasive case had no soft tissue complications, showing significant advantages.

Combined with the difficulties brought by current surgical methods, for calcaneal tuberosity avulsion fractures, using minimally invasive Bunnell suture of the Achilles tendon and knot fixation with calcaneal bone tunnel reconstruction effectively counteracts the upward traction force of the Achilles tendon on the posterior calcaneal tuberosity. For types I and III fractures, re-using a puncture needle to penetrate the tendon-bone junction and pass a suture to pull down the smaller bone fragments can compensate for the difficulty of screw fixation. This innovative technique can be flexibly switched between minimally invasive and open approaches. Compared with tension band fixation, this minimally invasive technique can avoid the impact of needle ends and steel wires on soft tissues, and also makes up for the difficulty of fixing small bone fragments with cannulated screws. For the challenges posed by poor skin conditions to anchor fixation and suture bridge techniques, the minimally invasive technique is an advantage. In addition, anchor suturing often requires the Krachow suture technique. Although this method has reliable tendon braiding, it requires a larger incision and multiple locking sutures, causing greater damage to the blood supply of the Achilles tendon (10). In contrast, we use Bunnell suture under minimally invasive conditions to pull the Achilles tendon, protecting its blood supply while ensuring strong tensile strength, without worrying about anchor loosening. Literature indicates that under the same material conditions, Bunnell suture and Krachow suture have equivalent tensile strength (11). There is also no need to consider the plantar fascia pain caused by side-locking loop suture (SLLS) and Tightrope technique (4, 12). This technique is also a feasible method for “beak” fractures and their variant fractures (as in the case in this article), combined with screws and limited incision fixation.

This study is a technical report, including only 2 patients, with a limited sample size and no concurrent control group, so it is unable to objectively confirm the advantages of this procedure over traditional procedures through inter-group comparison. The minimally invasive approach has the potential risk of sural nerve injury, and open surgery has hidden risks related to poor wound healing; research on these two types of complications still requires expanded sample sizes for further investigation.

There are objective defects in radiographic follow-up. This study is a retrospective analysis; for some patients, preoperative imaging from other hospitals could not be retrieved; patients who underwent open surgery did not complete the routine review at 3 months postoperatively, and only relied on immediate postoperative, 6-month postoperative, and 1-year postoperative imaging for longitudinal assessment; patients who underwent minimally invasive surgery only completed preoperative and 1-year postoperative imaging assessments, lacking imaging evidence from the transitional postoperative period.

The average follow-up duration of this group of cases is only 1 year; for delayed complications such as traumatic subtalar arthritis and long-term degeneration of the Achilles tendon, it is still necessary to extend the follow-up period for continuous observation.

This study only involves two patients, and there is no control group, which is a technical report. Limited by a very small sample size and a short-term follow-up of only one year, the evidence level of the study is low; therefore, it is impossible to draw reliable and universally applicable clinical conclusions, and it is impossible to prove that the improved technology has obvious advantages over other fixed methods. The technical advantages described in the article are only based on the preliminary observation of these two cases, and cannot be directly generalised to all cases of avulsion fractures of the bone nodules. In the future, it is necessary to carry out a larger sample study to verify the clinical value and scope of application of this technology.

Even with the above shortcomings, the current short-term follow-up results suggest that this modified suture combined with calcaneal tunnel fixation may show potential clinical value for the treatment of calcaneal tuberosity avulsion fractures, which are difficult to manage.

## Data Availability

The original contributions presented in the study are included in the article/Supplementary Material, further inquiries can be directed to the corresponding author.
